# Surgical treatment of the severe thoracic gastrocutaneous fistula by pedicled muscle flap filling and thoracoplasty after oesophagectomy for oesophageal squamous cell carcinoma: A case report

**DOI:** 10.1016/j.ijscr.2019.01.009

**Published:** 2019-01-24

**Authors:** Dongmin Yu, Zizi Zhou, Xiaoming Zhang

**Affiliations:** a Department of Thoracic and Cardiovascular Surgery, The First Affiliated Hospital of Gannan Medical University, Ganzhou, China; b Department of Cardio-Thoracic Surgery, Shenzhen University General Hospital, Shenzhen, China; c Department of Plastic and Reconstructive Surgery, BG Unfallklinik Ludwigshafen, University of Heidelberg, Heidelberg, Germany

**Keywords:** TGCF, thoracic gastrocutaneous fistula, TAF, thoracic anastomotic fistula, CT, computed tomography, SCM, sternocleidomastoid, Thoracic gastrocutaneous fistula, Muscle flap, Thoracoplasty, Oesophagectomy, Tracheostenosis, Case report

## Abstract

Background: Thoracic anastomotic fistula (TAF) is a severe postoperative complication of oesophagectomy, and its occurrence coupled with a thoracic gastrocutaneous fistula (TGCF) and tracheostenosis is very unusual and may lead to a fatal consequence.

Case presentation: We describe a case of an old female diagnosed with mid-oesophageal carcinoma, who presented with a TAF after oesophagectomy, which was healed by an effective treatment, while a severe TGCF and tracheostenosis appeared one month postoperation. The complications were detected by gastroscopy, barium oesophagogram and thoracic computed tomography (CT). Through surgical treatments, including pedicled muscle flap filling and thoracoplasty, and a correlated corrective procedure, the patient completely recovered and was discharged six months after the admission.

Conclusion: Treatment by pedicled muscle flap filling and thoracoplasty after oesophagectomy for oesophageal squamous cell carcinoma can be a curative alternative for the severe thoracic gastrocutaneous fistula.

## Highlights

• Effective treatment option for anastomotic leakage after oesophagectomy is controversial and full of difficulties at present.
• Patients with thoracic gastrocutaneous fistula and tracheostenosis are very unusual and may lead to a fatal consequence.
• Intrathoracic and cervical anastomotic leak and tracheostenosis appear in a same patient.
• Treatment by pedicled muscle flap filling and thoracoplasty could be a curative alternative for the severe thoracic gastrocutaneous fistula.

## 1 Background

Thoracic anastomotic fistula (TAF) is a serious complication after oesophagectomy of oesophageal carcinoma, with a reported incidence of 3–25% and a mortality rate of 30–60% [1]. When TAF is encountered, it can cause a longer hospital stay and increase mortality. TAF coupled with a thoracic gastrocutaneous fistula (TGCF) and tracheostenosis is very unusual and may lead to a fatal consequence for patients. At present, an effective treatment option for this complication is controversial. We describe here an improved surgical method for treating a severe thoracic gastrocutaneous fistula by pedicled muscle flap filling and thoracoplasty. This work is reported in line with the SCARE criteria [2].

## 2 Case presentation

**Fig. 1:**
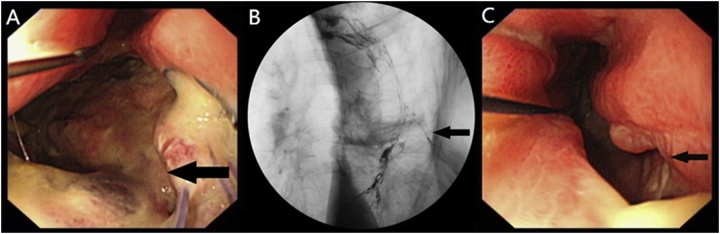
(A) Gastroscopy showing a fistula in the thoracic anastomosis; (B) Barium oesophagogram showing an outflow of the contrast agent from the thoracodorsal sinus tract; (C) Gastroscopy showing a gastroesophageal anastomotic fistula.

**Fig. 2:**
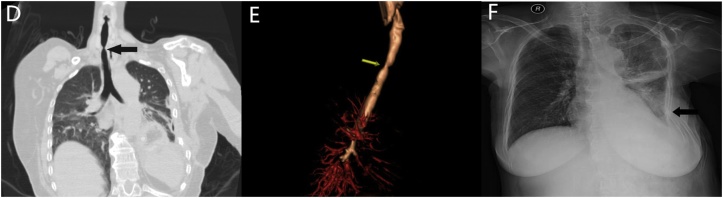
(D) and (E) CT showing the occurrence of tracheostenosis; (F) X-ray showing chest collapse after thoracoplasty and a normal tracheal morphology.

A 65-year-old female who suffered from progressive dysphagia for six months was admitted to our hospital with the diagnosis of mid-oesophageal carcinoma. Subsequently, she underwent left thoracotomy, oesophagectomy and intrathoracic oesophagogastrostomy (anastomotic stoma located in the cupula pleurae above the top of the aortic arch; pathological examination: moderately differentiated squamous cell carcinoma, invading the tunica adventitia of the oesophagus, with negative upper and lower incisal margins; staging: pT3N1M0). On the 11th postoperative day (subsequent days refer to the first operation), she began to vomit foul-smelling gastric juice; gastroscopy found a thoracic anastomosis fistula, and the size of orificium fistulae accounted for 1/3–1/2 of the anastomotic circumference (Fig. 1A). Therefore, a second surgery was performed on the 18th postoperative day to reanastomose the oesophagus and stomach in the neck. After this surgery, the patient presented with a cervical anastomotic fistula. One week later, thoracodorsal orificium fistulae, with a diameter of 2.5 cm, appeared in the first thoracic surgical incision, and approximately 400 ml of black gastric juice outflowed every day. On the 30th postoperative day, barium oesophagogram revealed that the contrast agent outflowed from the thoracodorsal sinus tract (Fig. 1B), and gastroscopy confirmed a 10-cm long longitudinal gastric fissure (approximately 20–30 cm away from the patient’s incisor), which appeared on the greater curvature side (Fig. 1C). After effective drainage, dressing changes and positive anti-infection measures, the cervical anastomotic fistula healed, while the patient gradually developed respiratory dyspnoea. On the 80th postoperative day, CT and fibreoptic bronchoscopy found a bound tracheostenosis located in the midtrachea (Fig. 2D, E). When thoracic cavity infection was limited and respiratory dyspnoea was relieved, a third surgery was performed, on the 90th postoperative day, to correct the tracheostenosis, repair the thoracic gastric fissure by filling it with a pedicled muscle flap, partly resect ribs 4–7, and close the thoracodorsal orificium fistula and vomicae (Fig. 2F). Afterwards, adequate nutrition support, thoracic washing and effective chest drainage, dressing changes, improved anti-infection control and other comprehensive treatments were carried out. The patient completely recovered and was discharged six months after admission.

## 3 Discussion

Oesophagectomy remains the gold standard curative therapy for the treatment of oesophageal cancer [3], even though it carries a risk of complications, which mainly include: pulmonary complications [4] and anastomotic leak [5].

The respiratory complications consist of pneumonia (21.4%), reintubation (16.2%), and ventilator dependency for more than 48 h [6]. Diminished airway protection including impaired swallowing, delayed gastric emptying and malnutrition in the perioperative period contributes to respiratory complications in patients undergoing an oesophagectomy [6,7]. Recognition of patients at risk for aspiration, adequate nutritional support and preoperative preparation including improving cardio-pulmonary function and cleaning intestinal tract would be of crucial importance in this regard. The tracheostenosis of this patient belong to such complication and should be corrected with surgical intervention.

As for anastomotic leak, several variables are implicated in the ultimate integrity of the anastomosis: anastomotic technique, anastomotic location, conduit location, and conduit selection [6]. Anastomotic techniques include hand sewn (single or double layer) and stapled (circular or linear). Cervical anastomosis does appear to be associated with a higher leak rate, which may cause a higher rate of postoperative stricture and dysphagia. However, it can limit the resultant surrounding contamination, while the thoracic anastomosis would result in a higher morbidity of pleural and mediastinal soilage [6], that’s the reason why we reanastomosed the oesophagus and stomach in the neck during the second operation. Regarding the conduit location, it’s better to place the neo-esophagus in the posterior mediastinum than in the retrosternal route because of a lower anastomotic leak rate, which attributable to reduced length requirements for the conduit and compression [8,9]. Conduit choice may also affect anastomotic integrity, although most surgeons presently prefer the stomach because of its ease of preparation, a robust blood supply, and sufficient length. Careful attention to surgical technique with regard to conduit perfusion, conduit tunneling, construction of the anastomosis, and gastric outlet procedures in addition to avoidance of laryngeal nerve injury and disruption of conduit blood supply all remain vital considerations [6]. Thus, the surgical options of oesophagectomy greatly depend on surgeon’s experience and awareness of outcomes.

As a severe post-oesophagectomy complication, anastomotic leak can cause serious infections, leading to electrolyte, acid-base, and nutrition supply imbalances, which cause longer hospital stays (mean length of stay: 27.4 days) and increase mortality (30-day mortality: 10%) [10,11]. Effective treatment options for these leaks are controversial and difficult to perform.

For patients with esophagogastric anastomotic leakage and serious infection, the traditional treatment is to perform thoracotomy again, remove the infected tissue, repair the orificium fistulae, improve anti-infective ability, provide adequate nutritional support and other treatments. However, the direct repair of the fistula was mostly unsuccessful due to the oedema of the anastomotic stoma. Therefore, conservative treatment of such anastomotic leakage is accepted to a certain extent at present. Moreover, Ye et al. explained that anastomotic leak following surgery for esophageal carcinoma should be treated individually based on the onset time, location, size, and extent of the leakage; and conservative treatment was still a safe and effective method [12].

In addition, the endoscopic treatment of thoracic anastomotic leakages by the placement of a covered metallic stent [[13], [14], [15]], an OTS clip or applying combined therapy after oesophagectomy for esophageal carcinoma is effective and safe [16]. And the median time of those treatment to achieve a complete healing from the diagnosis of the fistula or leakage was 44 days [16]. The disadvantages of such therapies include limited effectiveness (50–100%) and a certain malposition, as well as migration rate (10–42%) [[16], [17], [18], [19], [20], [21], [22]].

Studies also found endoscopic trans-fistula negative pressure drainage was reliable to promote fistula closure for post-oesophagectomy gastroesophageal anastomotic leak [23,24]. Han et al. revealed early diagnosis and treatment, nutritional supports were key to reduce mortality of gastrointestinal fistula [25]. Bhat et al. found the pedicled omental transposition for reinforcing the anastomotic suture line significantly reduced the incidence of leakage after esophagogastrectomy for carcinoma of the esophagus [26,27]. Nevertheless, any patient who is unstable or clinically deteriorating should undergo operative therapy rather than stenting or conservative treatment.

In this case, intrathoracic oesophagogastrostomy was performed above the top of the aortic arch since this surgical approach has been proven to reduce some complications [28]. However, TAF developed 11 days after this first surgery. The possible reasons included malnutrition of the anastomotic stoma, separation of the anastomotic nail from its functional position, postoperative infection among other possible predisposing factors. Because gastroscopy confirmed that the anastomotic fistula was large, with few chances of self-healing, and was coupled with serious thoracic cavity infection, which could endanger the patient's life, we moved the anastomotic stoma to the neck. Unfortunately, a neck anastomotic fistula appeared after this operation. A strong cervical anastomotic tension, caused by a limited gastric remnant and inflammation, may lead to this problem. Meanwhile, TGCF and a thoracodorsal sinus tract also emerged, aetiologies of which are diverse, such as small stomach omentum vessel thrombosis, poor resistance to secondary infection, a slip of suture threads and the multiple operative wound, etc. It was not suitable to repair the gastric fissure immediately because of thoracic cavity infection, which would cause suture threads of the gastric fissure to drop and the gastric remnant to crack. By ways of conservative treatment, the neck anastomotic fistula healed, while tracheostenosis occurred, which was probably caused by inflammatory adhesion towards the tracheal membrane, local exudation and nodular hyperplasia, which correlated with the neck anastomotic inflammation and made the third operation indispensable. There are three advantages of using a pedicled muscle flap: (1) it can be conveniently created from intercostal muscle and the latissimus dorsi; (2) sufficient blood supply and the ability to resist infection make such flap an ideal tissue for repairing gastric orificium fistulae; and (3) it minimizes the lesion and saves operation time because the flap is able to be obtained nearby easily. Other flaps would not achieve all those goals above. In addition, thoracoplasty can help close vomicae. However, the disadvantages of such pedicled muscles flap include impairment of muscle integrity and original function, as well as increase of pains in corresponding chest wall. And thoracoplasty would lead to chest deformity, mediastinum displacement and decrease of lung volume.

This case is unique on account of a severe TAF, TGCF and other postoperative complications such as tracheostenosis. In the past, Moody et al. found the use of the sternocleidomastoid(SCM) flap helped repair laryngectomy and cervical oesophagectomy defects [29]. Nakajima et al. also revealed SCM flap repair was an effective and minimally invasive treatment method for cervical anastomotic leakage after oesophagectomy [30]. Heitmiller et al. found the pectoralis myocutaneous flap repair of cervical anastomotic complications including anastomotic strictures or leakage was safe and well tolerated [31]. Kiyotomi et al. [32] reported the use of a left pectoralis major muscle flap to fill the interspace between the trachea and gastric tube. Nevertheless, the treatment of TGCF with a pedicled muscle flap and thoracoplasty has not been reported previously. This case suggests that when treating older patients or patients with poor cardiopulmonary function, the method of cervical oesophagogastrostomy through the oesophageal bed should be recommended to reduce complications and mortality. Even if cervical anastomotic leaks are present, most of them can be successfully treated with conservative approaches or by gastroscopy [1,33]. And as compared with thoracic anastomotic leakage, cervical anastomotic leak is better tolerated and more easily treated by opening the cervical incision for drainage [34]. Furthermore, coverage of the omentum or pedicled greater omentum displacement is also a better way to prevent gastroesophageal anastomotic leakages. When severe TGCF occurs, surgical treatments by pedicled muscle flap(intercostal muscle and the latissimus dorsi) filling and thoracoplasty can be alternatives.

A follow-up investigation by telephone was carried out several times, and the patient could freely eat food and had no uncomfortable symptoms, even though she did not have additional gastroscopy because of a fear of this examination. The lack of objective image information after the patient was discharged was the limitation of our study.

## 4 Conclusion

Oesophagectomy is a standard and effective surgical treatment for oesophageal carcinoma. Esophagogastric anastomotic leakage is a life-threatening postoperative complication. TAF coupled with TGCF and tracheostenosis is very unusual and may lead to a fatal consequence, and treatment by pedicled muscle flap(intercostal muscle and the latissimus dorsi) filling and thoracoplasty after oesophagectomy for oesophageal squamous cell carcinoma can provide a curative alternative for the severe thoracic gastrocutaneous fistula.

## Conflicts of interest

All authors report no conflicts of interest.

## Funding

This study was supported by a grant from the China Scholarship Council (No. 201708080131to Zizi Zhou).

## Ethics approval

The present study is exempt from ethical approval in our institution.

## Consent

Written informed consent was obtained from the patient for publication of this case report and accompanying images, and the identity of the patient has been protected.

## Author contribution

Dongming Yu, Zizi Zhou and Xiaoming Zhang: paper concept, design, data collection and interpretation. Dongming Yu and Zizi Zhou: writing the manuscript. Xiaoming Zhang: reviewing and editing. Dongming Yu was the operating surgeon and responsible for drafting the article content, and Xiaoming Zhang in charge of revising the article content and the final approval of the manuscript prior to submission.

## Registration of research studies

Not applicable.

## Guarantor

Dongming Yu and Zizi Zhou.

## Provenance and peer review

Not commissioned, externally peer-reviewed.

## References

[1] Turkyilmaz A., Eroglu A., Aydin Y., Tekinbas C., Muharrem Erol.M., Karaoglanoglu N. (2009). The management of esophagogastric anastomotic leak after esophagectomy for esophageal carcinoma. Dis. Esophagus.

[2] Agha R.A., Fowler A.J., Saetta A., Barai I., Rajmohan S., Orgill D.P., for the SCARE Group (2016). The SCARE statement: consensus-based surgical case report guidelines. Int. J. Surg..

[3] Depypere L., Coosemans W., Nafteux P., Van VeerH., Neyrinck A., Coppens S., Boelens C., Laes K., Lerut T. (2017). Video-assisted thoracoscopic surgery and open chest surgery in esophageal cancer treatment: present and future. J. Vis. Surg..

[4] Pisarska M., Małczak P., Major P., Wysocki M., Budzyński A., Pędziwiatr M. (2017). Enhanced recovery after surgery protocol in oesophageal cancer surgery: systematic review and meta-analysis. PLoS One.

[5] Halliday L.J., Markar S.R., Doran S.L.F., Moorthy K. (2017). Enhanced recovery protocols after oesophagectomy. J. Thorac. Dis..

[6] Raymond D. (2012). Complications of esophagectomy. Surg. Clin. North Am..

[7] Martin R.E., Letsos P., Taves D.H. (2001). Oropharyngeal dysphagia in esophageal cancer before and after transhiatal esophagectomy. Dysphagia.

[8] Orringer M.B., Marshall B., Chang A.C., Lee J., Pickens A., Lau C.L. (2007). Two thousand transhiatal esophagectomies: changing trends, lessons learned. Ann. Surg..

[9] Collard J.M., Tinton N., Malaise J., Romagnoli R., Otte J.B., Kestens P.J. (1995). Esophageal replacement: gastric tube or whole stomach?. Ann. Thorac. Surg..

[10] Kassis E.S., Kosinski A.S., Ross P., Koppes K.E., Donahue J.M., Daniel V.C. (2013). Predictors of anastomotic leak after esophagectomy: an analysis of the society of thoracic surgeons general thoracic database. Ann. Thorac. Surg..

[11] Worrell S.G., Chang A.C. (2017). Risk adjustment and performance measurement for esophageal cancer resection. Thorac. Surg. Clin..

[12] Ye H.Y., Huang W.Z., Wu Y.M., Liang Y., Zheng J.M., Jiang H.M. (2012). Personalized management of anastomotic leak after surgery for esophageal carcinoma. Chin. Med. Sci. J..

[13] Schubert D., Scheidbach H., Kuhn R., Wex C., Weiss G., Eder F., Lippert H., Pross M. (2005). Endoscopic treatment of thoracic esophageal anastomotic leaks by using silicone-covered, self-expanding polyester stents. Gastrointest. Endosc..

[14] Giri A.K., Bassi K.K., Gupta V.K., Singh B.P., Abraham S.W., Pandey K.K. (2015). Esophageal stent placement for acute intra-thoracic anastomotic leak after esophagectomy. Indian J. Cancer.

[15] Kauer W.K., Stein H.J., Dittler H.J., Siewert J.R. (2008). Stent implantation as a treatment option in patients with thoracic anastomotic leaks after esophagectomy. Surg. Endosc..

[16] Gonzalez J.M., Servajean C., Aider B., Gasmi M., D’Journo X.B., Leone M., Grimaud J.C., Barthet M. (2016). Efficacy of the endoscopic management of postoperative fistulas of leakages after esophageal surgery for cancer: a retrospective series. Surg. Endosc..

[17] Zisis C., Guillin A., Heyries L., Lienne P., D’Journo X.B., Doddoli C., Giudicelli R., Thomas P.A. (2008). Stent placement in the management of oesophageal leaks. Eur. J. Cardiothorac. Surg..

[18] Gonzalez J.M., Garces Duran R., Vanbiervliet G., Lestelle V., Gomercic C., Gasmi M., Desjeux A., Grimaud J.C., Barthet M. (2015). Double-type metallic stents efficacy for the management of post-operative fistulas, leakages, and perforations of the upper gastrointestinal tract. Surg. Endosc..

[19] van Boeckel P.G., Dua K.S., Weusten B.L., Schmits R.J., Surapaneni N., Timmer R., Vleggaar F.P., Siersema P.D. (2012). Fully covered self-expandable metal stents (SEMS), partially covered SEMS and self-expandable plastic stents for the treatment of benign esophageal ruptures and anastomotic leaks. BMC Gastroenterol..

[20] Kauer W.K., Stein H.J., Dittler H.J., Siewert J.R. (2008). Stent implantation as a treatment option in patients with thoracic anastomotic leaks after esophagectomy. Surg. Endosc..

[21] Babor R., Talbot M., Tyndal A. (2009). Treatment of upper gastrointestinal leaks with a removable, covered, self-expanding metallic stent. Surg. Laparosc. Endosc. Percutan. Tech..

[22] Salminen P., Gullichsen R., Laine S. (2009). Use of self-expandable metal stents for the treatment of esophageal perforations and anastomotic leaks. Surg. Endosc..

[23] Liu Y.N., Yan Y., Li S.J., Liu H., Wu Q., Zhang L.J., Yang Y., Chen J.F. (2014). Reliable management of post-esophagectomy anastomotic fistula with endoscopic trans-fistula negative pressure drainage. World J. Surg. Oncol..

[24] Zhu Z., Li Z., He Z., Wang Y. (2017). Endoscopic trans-fistula drainage for gastroesophageal anastomotic fistula with para-fistula abscess after esophagectomy. Zhejiang Da Xue Xue Bao Yi Xue Ban.

[25] Han Y., Zhao H., Xu H., Liu S., Li L., Jiang C., Yang B. (2014). Cure and prevention strategy for postoperative gastrointestinal fistula after esophageal and gastric cardiac cancer surgery. Hepatogastroenterology.

[26] Bhat M.A., Dar M.A., Lone G.N., Dar A.M. (2006). Use of pedicled omentum in esophagogastric anastomosis for prevention of anastomotic leak. Ann. Thorac. Surg..

[27] Sepesi B., Swisher S.G., Walsh G.L., Correa A., Mehran R.J., Rice D., Roth J., Vaporciyan A., Hofstetter W.L. (2012). Omental reinforcement of the thoracic esophagogastric anastomosis: an analysis of leak and reintervention rates in patients undergoing planned and salvage esophagectomy. J. Thorac. Cardiovasc. Surg..

[28] Usui H., Fukaya M., Itatsu K., Miyata K., Miyahara R., Funasaka K., Nagino M. (2018). The impact of the location of esophagogastrostomy on acid and duodenogastroesophageal reflux after transthoracic esophagectomy with gastric tube reconstruction and intrathoracic esophagogastrostomy. W. J. Surg..

[29] Moody L., Hunter C., Nazerali R., Lee G.K. (2016). The use of the sternocleidomastoid flap helps reduce complications after free jejunal flap reconstructions in total laryngectomy and cervical esophagectomy defects. Ann. Plast. Surg..

[30] Nakajima M., Satomura H., Takahashi M., Muroi H., Kuwano H., Kato H. (2014). Effectiveness of sternocleidomastoid flap repair for cervical anastomotic leakage after esophageal reconstruction. Dig. Surg..

[31] Heitmiller R.F., McQuone S.J., Eisele D.W. (1998). The utility of the pectoralis myocutaneous flap in the management of select cervical esophageal anastomotic complications. J. Thorac. Cardiovasc. Surg..

[32] Maruyama K., Motoyama S., Okuyama M., Sato Y., Hayashi K., Minamiya Y., Ogawa J. (2007). Esophagotracheal fistula caused by gastroesophageal reflux 9 years after esophagectomy. W. J. Gastroenterol..

[33] Larburu E.S., Gonzales R.J., Elorza O.J.L., Asensio G.J.I., Diez d.Val.I., Eizaguirre L.E., Mar M.B. (2013). Cervical anastomotic leak after esophagectomy: diagnosis and management. Cir. Esp..

[34] Ng T., Vezeridis M.P. (2010). Advances in the surgical treatment of esophageal cancer. J. Surg. Oncol..

